# Self-stigma in serious mental illness and autism spectrum disorder: Results from the REHABase national psychiatric rehabilitation cohort

**DOI:** 10.1192/j.eurpsy.2019.12

**Published:** 2020-02-07

**Authors:** J. Dubreucq, J. Plasse, F. Gabayet, M. Faraldo, O. Blanc, I. Chereau, S. Cervello, G. Couhet, C. Demily, N. Guillard-Bouhet, B. Gouache, N. Jaafari, G. Legrand, E. Legros-Lafarge, R. Pommier, C. Quilès, D. Straub, H. Verdoux, F. Vignaga, C. Massoubre, REHABAse Network, N. Franck

**Affiliations:** 1 Centre de Neurosciences Cognitive, UMR 5229, CNRS & Université Lyon 1, Lyon, France; 2 Centre Référent de Réhabilitation Psychosociale et de Remédiation Cognitive (C3R), Centre Hospitalier Alpes Isère, Grenoble, France; 3 Fondation FondaMental, Créteil, France; 4 Réseau Handicap Psychique, Grenoble, France; 5 Centre Référent Lyonnais de Réhabilitation Psychosociale CL3R, Centre Hospitalier Le Vinatier, Lyon, France; 6 Centre Ressource de Réhabilitation Psychosociale et de Remédiation Cognitive, Hôpital Le Vinatier, UMR 5229, CNRS & Université Lyon 1, Université de Lyon, Lyon, France; 7 Centre Hospitalier Sainte Marie de Clermont Ferrand, 63037 Clermont-Ferrand Cedex 1, France; 8 Centre Référent de Réhabilitation Psychosociale C2RP Nouvelle-Aquitaine Sud, Pôle de Réhabilitation Psychosociale, Centre de la Tour de Gassies, Bruges, France; 9 Centre de Référence Maladies Rares Génopsy, Centre Hospitalier Le Vinatier, UMR 5229, CNRS & Université Lyon 1, Université de Lyon, Lyon, France; 10 CREATIV & URC Pierre Deniker, CH Laborit, Poitiers, France; 11 Centre Référent de Réhabilitation Psychosociale de Limoges C2RL, CH Esquirol, Limoges, France; 12 REHALise, CHU de Saint-Etienne, Saint-Priest-en-Jarez, France; 13 Centre Référent de Réhabilitation Psychosociale C2RP Nouvelle Aquitaine Sud, Pôle Universitaire de Psychiatrie Adulte, Centre Hospitalier Charles Perrens, Bordeaux & Univ. Bordeaux, INSERM, Bordeaux Population Health Research Center, Team Pharmacoepidemiology, UMR 1219, 33000 Bordeaux, France; 14 Centre de Réhabilitation Psychosociale, Centre Hospitalier de Roanne, Roanne, France; 15 Dispositif de Soins de Réhabilitation Psychosociale, Centre Psychothérapeutique de l’Ain, Bourg-en-Bresse, France; 16 CMP B, CHU, EA 7280 Faculté de Médecine, Université d’Auvergne, BP 69 63003 Clermont-Ferrand Cedex 1, France

**Keywords:** Self-stigma, serious mental illness, autism spectrum disorders, prevalence, psychiatric rehabilitation

## Abstract

**Background.:**

Self-stigma is a major issue in serious mental illness (SMI) and is negatively associated with patient outcomes. Most studies have been conducted in schizophrenia (SZ). Less is known about self-stigma in other SMI and autism spectrum disorder (ASD). The objectives of this study are: (i) to assess the frequency of self-stigma in a multicentric nonselected psychiatric rehabilitation SMI and ASD sample; and (ii) to investigate the correlates of elevated self-stigma in different SMI conditions and in ASD.

**Methods.:**

A total of 738 SMI or ASD outpatients were recruited from the French National Centers of Reference for Psychiatric Rehabilitation cohort (REHABase). Evaluations included sociodemographic data, illness characteristics, and standardized scales for clinical severity, quality of life, satisfaction with life, wellbeing, personal recovery, a large cognitive battery, and daily functioning assessment.

**Results.:**

31.2% of the total sample had elevated self-stigma. The highest prevalence (43.8%) was found in borderline personality disorder and the lowest (22.2%) in ASD. In the multivariate analysis, elevated self-stigma was best predicted by early stages of personal recovery (moratorium, *p* = 0.001, OR = 4.0 [1.78–8.98]; awareness, *p* = 0.011, OR = 2.87 [1.28–6.44]), history of suicide attempt (*p* = 0.001, OR = 2.27 [1.37–3.76]), insight (*p* = 0.002, OR = 1.22 [1.08–1.38]), wellbeing (*p* = 0.037, OR = 0.77 [0.60–0.98]), and satisfaction with interpersonal relationships (*p* < 0.001, OR = 0.85 [0.78–0.93]).

**Conclusions.:**

The present study has confirmed the importance of addressing self-stigma in SMI and ASD patients enrolled in psychiatric rehabilitation. The effectiveness of psychiatric rehabilitation on self-stigma and the potential mediating effects of changes in self-stigma on treatment outcomes should be further investigated.

## Introduction

Many members of the general public endorse negative stereotypes about Serious Mental Illness (SMI) or autism spectrum disorders (ASD). These include expectations of violence and an inability to work or to live in society that can lead to social distancing and rejection. Although less stigmatized than schizophrenia (SZ), ASD was associated with dangerousness to self and an inability to work in over 20% of the 1,000 respondents in a 2012 French population survey [1]. Most people with SMI are aware of these stereotypes and expect to be discriminated against by other people because of their condition (69.4% of the 1,229 participants with SZ and 71.6% of the 1,182 participants with mood disorders in the GAMIAN-Europe study had high perceived stigma [2,3]). Self-stigma—or internalized stigma (IS)—occurs when someone accepts the negative stereotypes about SMI or ASD as a true description of him/herself [4]. IS refers to the process wherein a person’s previously held social identity (defined by social roles such as son, brother, sister, friend, employee, or potential partner) is progressively replaced by a devalued and stigmatized view of oneself. IS is highly prevalent in Europe (41.7% in SZ, 21.7% in mood disorders [1,2]) and the United States (36.1% out of 144 people with SMI [5]). According to the “illness identity model” [6], IS can have pervasive effects on recovery-related outcomes, including self-esteem, hopefulness, wellbeing, motivation to achieve personal life goals, social interaction, employment, and symptom severity [7–10]. Individuals with elevated self-stigma report more dysfunctional attitudes, social withdrawal, depressive symptoms, and increased suicidal ideation [6,11,12]. Several studies also support that high insight into illness directly predicts and compounds the effects of self-stigma on depression [11,13,14]. Impaired cognitive functioning, metacognition, and social cognition predict increased self-stigma [14–17].

Stigma studies have mostly targeted individuals with SZ (54.3% out of 127 articles; Livingston and Boyd [18]; 42.3% of 220 articles in a recent review [19]), SMI (40% of 220 articles [19]) or mood disorders (bipolar disorder [BD] 5% and major depression [MDD] 5.7% of 220 articles [19]). Considerably less is known about IS in borderline personality disorder (BPD), anxiety disorders, and ASD. Some studies found that self-stigma was higher in BPD than in SZ (Internalized Stigma of Mental Illness [ISMI] mean total score = 2.43 BPD vs. 2.34 SZ [20]) and lower in anxiety disorders (ISMI total score ranging from 1.98 to 2.24 in Europe [19]) or ASD (ISMI total = 1.93, 15.7% out of 149 participants showing elevated self-stigma [21]). Self-stigma was also associated with increased psychiatric symptoms, reduced hope, and lower treatment adherence in anxiety disorders or BPD [20,22,23]. The correlates of IS in ASD are still unknown. Several potential predictors were identified, the most significant of which were insight into illness, hopelessness, impaired cognitive functioning, avoidant coping strategies, stigma stress, perceived stigma, and psychiatric symptoms [19]. Contrasting results were found for occupational status, psychiatric diagnosis, and illness duration. Individual studies identified other factors such as parenting status or decreased wellbeing as potential predictors of self-stigma [24,25]. Few studies investigated self-stigma within the context of psychiatric rehabilitation or recovery-oriented practices (7.7% of 220 articles [19]). Recovery-oriented interventions have shown preliminary effectiveness on self-stigma [26–29]. Self-stigma was associated with worse treatment outcomes during vocational rehabilitation [30]. However, the prevalence of IS in patients attending to psychiatric rehabilitation and its effects on therapeutic outcomes remain largely unknown. To the best of our knowledge, only one study, of a small sample of SZ patients, has investigated self-stigma in France, finding moderate levels of IS (*n* = 62, ISMI total score = 2.23 [31]). The frequency of elevated self-stigma in SMI or ASD is still unknown.

To sum up, self-stigma is a major issue in SMI and is associated with poor clinical and functional outcomes. Its frequency in SZ or mood disorders is high in European countries. Considerably less is known about self-stigma in other SMI and in ASD and there are no data on IS prevalence in France. Previous research indicates that insight into illness, psychiatric symptoms, and cognitive functioning could predict the level of self-stigma. Other variables from individual studies such as parenting status or wellbeing might predict IS but these results need to be confirmed. The correlates of IS in BPD, anxiety disorders and ASD remain largely unknown.

The objectives of the present study were: (i) to assess the frequency of self-stigma in a multicentric nonselected psychiatric rehabilitation SMI and ASD sample; and (ii) to investigate the correlates of elevated self-stigma in different SMI conditions and in ASD.

## Materials and Methods

### Study population

The REHABase cohort is made up of patients from a French network of psychiatric rehabilitation centers that has been extensively described in a previous article [32]. Patients are referred to these centers by their general practitioner or psychiatrist, who remains in charge of routine care and treatment. The inclusion criteria are: (i) a diagnosis of SMI (i.e., SZ, BD, BPD, MDD, or severe anxiety disorders, according to the SAMSAH 2013 definition [33]) or ASD (DMS-5 criteria [34]); (ii) a score below the cut-off scores for social recovery according to Jääskeläinen et al. in 2013 (a score of less than 61 on the Global Assessment of Functioning (GAF) Scale [35]). A comprehensive clinical, functional, and cognitive assessment is performed to establish the individual’s strengths and weaknesses, autonomy, and occupational level. Therapeutic tools are selected based on the participant’s personal life goals as part of an individualized psychiatric rehabilitation action plan. The action plan can include psychoeducation, joint crisis plans, cognitive remediation, cognitive behavior therapy, social skills training, peer-delivered interventions, and supported employment [32]. Follow-up is planned to last for 2 to 3 years. Evaluations are scheduled at baseline, annually, and after the action plan is completed. The action plan can begin before the evaluation when clinically relevant to support patient’s engagement in mental health care or psychiatric rehabilitation. This is for instance the case for strengths-based case management in early psychosis and supported employment, housing, or parenting. Two thousand and fifty-three patients were included in the eight REHABase sites between January 2016 and April 2019. Seven hundred and thirty-eight (35.9%) were effectively evaluated at the time of extraction. The study obtained the authorizations required under French legislation (French National Advisory Committee for the Treatment of Information in Health Research, 16.060bis; French National Computing and Freedom Committee, DR-2017-268). All participants gave their informed consent.

### Site selection and training

Site selection and training have been described in a previous article [32]. Since this first study, three new sites have joined the REHABase network. The eight sites (Lyon, Grenoble, Saint-Etienne, Limoges/Poitiers, Bordeaux, Clermont-Ferrand, Roanne, and Bourg-en-Bresse) opened in France were already actively involved in the treatment of patients with SMI (pharmacological and nonpharmacological), as well as research on psychiatric rehabilitation and recovery-oriented practices. Each center has accepted and been trained to use the same package of assessment tools for the baseline visit and follow-up. Clinical team members have regular group meetings to monitor quality control and ensure good inter-rater reliability.

### Data collected

General information on education, marital status, economic status, illness onset and trajectory and comorbidities was recorded. Self-stigma was assessed using the ISMI Scale [2,36], a 29-item self-report measure designed to assess people’s personal experience of stigma related to mental disorders and is rated on a 4-point Likert Scale. Items are summed to provide a mean total score and five subscale scores (alienation or feeling of being a devalued member of society; stereotype endorsement or agreement with negative attitudes about SMI; experience of discrimination; and social withdrawal as a coping strategy and stigma resistance). Stigma resistance is generally excluded because of poor correlations with other subscales [37]. A higher score reflects a higher level of self-stigma. A score above 2.5 indicates a moderate to high level of self-stigma [2,3]. Satisfaction in four life dimensions (social, familial, and intimate relationships, occupational status) was measured using visual analogue scales and a structured interview adapted from the Client Assessment of Strengths, Interests, and Goals (CASIG) [38]. Illness severity was assessed using the Positive and Negative Syndrome (PANSS) [39] and the Clinical Global Impression (CGI) [40] scales. Insight and treatment adherence were measured with self-reported measures (Birchwood Insight Scale [BIS] [41]; Medication Adherence Rating Scale [MARS] [42]). General functioning was measured with the GAF Scale [43]. Quality of Life was evaluated with the self-reported Subjective Quality of Life Scale (S-QoL) [44] and wellbeing using the Warwick-Edinburgh Mental Well-being Scale (WEMWBS) [45]. Self-esteem was assessed with the Self-Esteem Rating Scale-Short Form (SERS-SF) [46] and personal recovery was measured using the self-reporting Stages of Recovery Instrument (STORI) [47]. Baseline neuropsychological cognitive assessments include the Wechsler Adult Intelligence Scale-4th edition (WAIS-IV) [48] subscale assessing short-term and working memory, the California Verbal Learning Test [49] or RL/RI-16 [50] for global verbal memory, d2-R for selective attention, concentration and speed of processing [51] and the shopping test [52] or Six Element Test [53] for planning abilities. Theory of mind was assessed using the Movie for the Assessment of Social Cognition [54] and attribution style with the Ambiguous Intentions and Hostility Questionnaire (AIHQ) [55].

### Statistical analysis

Data are presented as the mean and SD for continuous variables and number and percentage for categorical variables. A one-way analysis of variance was performed and the *p*-values adjusted for multiple comparisons using Tukey’s method. The internal consistency of the ISMI total scale and subscales was measured using Cronbach’s alpha (*α*).

Patients’ baseline characteristics were analyzed to identify factors associated with a high level of self-stigma. Categorical variables were analyzed using chi-square analysis or Fisher’s exact test as appropriate, and continuous variables were analyzed using Student’s *t* test or Wilcoxon’s test for non-normal variables. Logistic regression was used to calculate OR with 95% CI. Finally, a multivariate logistic regression with stepwise selection was performed on all predictors to investigate the factors independently associated with the level of self-stigma. The collinearity was checked using the variance inflation factor. *p*-Values <0.05 were considered significant. All statistical analyses were performed using R (R Foundation for Statistical Computing, Vienna, Austria; https://www.R-project.org/) [56].

## Results

Seven hundred and thirty-eight clinically stabilized patients were recruited from the REHABase network. They were included in this cohort study between January 2016 and April 2019. SZ was the most common diagnosis with 466 patients (63.1%). Other diagnoses were BD (117, 15.9%), BPD (64, 8.7%), ASD (45, 6.1%), MDD (27, 3.7%), and anxiety disorders (19, 2.5%). The included patients were mostly men (500, 67.8%), with a mean duration of illness of 11.5 (SD = 8.7) years and a mean baseline PANSS total score of 66.4 (SD = 19.1). Baseline sample characteristics are shown in Table 1. There were significant differences according to participant’s psychiatric diagnosis (data not shown). The analysis of variance showed that diagnosis had a significant effect on GAF (*F*(5, 514) = 3.71, *p* = 0.03). The post hoc analyses indicated that global functioning was lower in SZ than in BD (diff = −6.3, *p* = 0.02). A significant effect was also found on subjective QoL (*F*(5,649) = 4.64, *p* < 0.001). SQoL was significantly lower in anxiety disorders (diff = −13.5, *p* = 0.02) and BPD (diff = −8.4, *p* = 0.01) compared with SZ. Wellbeing (*F*(5, 699) = 3.28, *p* = 0.006) and satisfaction with interpersonal relationships (*F*(5, 569) = 5.48, *p* < 0.001) were significantly lower in BPD compared with SZ and BD (diff = −0.6 for WEMWBS and diff = −2 for satisfaction with interpersonal relationships).

**Table 1.** Patient characteristics

		All patients (*n* = 738)
Gender (*n* = 738)	Male	500 (67.8)
	Female	238 (32.2)
Age at the time of admission (*n* = 738)	Mean (SD)	33.2 (10.1)
Education level (*n* = 726)	Primary/secondary school	137 (18.9)
	High school	365 (50.3)
	University	224 (30.8)
Housing status (*n* = 729)	Personal accommodation	362 (49.7)
	Family accommodation	282 (38.7)
	Others (supervised apartment, household)	85 (11.6)
Occupational status (*n* = 723)	Without income	149 (20.6)
	Competitive/sheltered work	62 (8.6)
	Unemployment/disability benefits	512 (70.8)
Marital status (*n* = 728)	Single	625 (85.9)
	In a couple	103 (14.1)
Number of children (*n* = 722)	Without child	606 (83.9)
	At least one child	116 (16.1)
Legal protection (*n* = 721)	Without	613 (85.0)
	With	108 (15.0)

Values are mean (SD) or *n* (%).

### Frequency of elevated self-stigma

The internal consistency for the 24-item ISMI was *α* = 0.90. The stigma resistance subscale had an internal consistency of *α* = 0.49. The following values were found for the other four subscales: alienation *α* = 0.81, stereotype endorsement *α* = 0.69, experience of discrimination *α* = 0.78, social withdrawal *α* = 0.78. Table 2 presents the results of grouping ISMI total and subscale scores for the total sample and per diagnosis, using minimal-low and moderate-high self-stigma categories. Elevated self-stigma was found in 31.2% of the total sample (29.8% in SZ, 29.9% in BD, 40.7% in MDD, 42.1% in anxiety disorders, 43.8% in BPD and 22.2% in ASD). The highest extent of self-stigma was found in BPD (mean ISMI total score = 2.36) and the lowest in ASD (2.13).

**Table 2.** Internalized stigma in psychiatric disorders in the study sample

Population (*n* = 738)	Full scale—mean (SD)		Subscales—mean (SD)				
	With stigma resistance	Without stigma resistance	Alienation	Stereotype endorsement	Discrimination experience	Social withdrawal	Stigma resistance
All diagnoses	2.25 (0.45)	2.2 (0.51)	2.48 (0.69)	1.9 (0.48)	2.17 (0.65)	2.31 (0.65)	2.54 (0.51)
Autism spectrum disorder (ASD; *n* = 45)	2.2 (0.42)	2.13 (0.48)	2.39 (0.65)	1.74 (0.42)	2.18 (0.65)	2.29 (0.65)	2.48 (0.52)
Schizophrenia (SZ; *n* = 466)	2.22 (0.46)	2.18 (0.51)	2.42 (0.7)	1.9 (0.49)	2.15 (0.64)	2.27 (0.63)	2.56 (0.51)
Bipolar disorder (BD; *n* = 117)	2.25 (0.44)	2.2 (0.53)	2.59 (0.67)	1.85 (0.47)	2.22 (0.68)	2.25 (0.67)	2.52 (0.53)
Major depressive disorder (MDD; *n* = 27)	2.31 (0.49)	2.29 (0.56)	2.61 (0.78)	1.95 (0.53)	2.21 (0.69)	2.44 (0.67)	2.58 (0.53)
Anxiety disorders (*n* = 19)	2.38 (0.48)	2.35 (0.56)	2.87 (0.71)	1.97 (0.44)	2.15 (0.74)	2.44 (0.68)	2.44 (0.38)
Borderline personality disorder (BPD; *n* = 64)	2.38 (0.42)	2.36 (0.47)	2.65 (0.62)	2.05 (0.42)	2.23 (0.59)	2.55 (0.68)	2.52 (0.48)

### Correlates of elevated self-stigma

Table 3 presents the results of the univariate analyses for the correlates of elevated self-stigma. Significant associations were found with female gender (*p* = 0.095; OR = 1.32 [0.95–1.83]) and older age at the time of admission (*p* = 0.002; OR = 1.03 [1.01–1.04]). Elevated self-stigma was positively associated with psychiatric comorbidities (*p* = 0.05, OR = 1.46 [1.00–2.12]), history of suicide attempt (*p* < 0.001, OR = 2.30 [1.64–3.22]), insight (BIS, *p* < 0.001, OR = 1.27 [1.17–1.38]) and clinical severity (CGI, *p* < 0.001, OR = 1.54 [1.28–1.87]). Elevated self-stigma was negatively associated with satisfaction with interpersonal (*p* < 0.001, OR = 0.79 [0.73–0.84]), familial (*p* < 0.001, OR = 0.85 [0.79–0.91]), and intimate relationships (*p* < 0.001, OR = 0.88 [0.83–0.93]). Negative associations were found between elevated self-stigma and wellbeing (WEMWBS total score, *p* < 0.001, OR = 0.54 [0.47–0.62]) or treatment adherence (MARS, *p* < 0.001; OR = 0.84 [0.77–0.92]). Elevated self-stigma was negatively associated with personal recovery (*p* < 0.001). Compared with participants in the growth stage of personal recovery, those in earlier stages showed significantly higher levels of self-stigma (moratorium, OR = 9.83 [5.73–17.33]; awareness, OR = 5.49 [3.08–9.97]; preparation, OR = 4.00 [2.06–7.79]; rebuilding, OR = 2.34 [1.34–4.15]). No significant correlations were found with age of onset, educational level, housing status, occupational status, duration of psychiatric hospitalizations, intimate relationships, parenting status, and cognitive functioning.

**Table 3.** Association between medical factors and quality of life with self-stigma in univariate logistic regression

		All patients (*n* = 738)	Normal self-stigma (*n* = 508)	Elevated self-stigma (*n* = 230)	*p*-Value	Odds ratio (univariate)
Diagnoses class (DSM5; *n* = 738)	Autism Spectrum Disorder (ASD)	45 (6.1)	35 (6.9)	10 (4.3)	0.1	
	Schizophrenia (SZ)	466 (63.1)	328 (64.6)	138 (60.0)		
	Bipolar disorder (BD)	117 (15.9)	82 (16.1)	35 (15.2)		
	Major depressive disorder (MDD)	27 (3.7)	16 (3.1)	11 (4.8)		
	Anxiety disorders	19 (2.6)	11 (2.2)	8 (3.5)		
	Borderline personality disorder (BPD)	64 (8.7)	36 (7.1)	28 (12.2)		
Illness duration (years; *n* = 649)	Mean (SD)	11.5 (8.7)	10.8 (8.3)	13.1 (9.3)	**0.001**	1.03 (1.01–1.05, *p* = 0.002)
Psychiatric comorbidity (*n* = 629)	No	465 (73.9)	333 (76.2)	132 (68.8)	0.05	–
	Yes	164 (26.1)	104 (23.8)	60 (31.2)		1.46 (1.00–2.12, *p* = 0.051)
Number of previous admissions (*n* = 667)	Mean (SD)	3.2 (3.6)	3.1 (3.5)	3.6 (3.8)	**0.04**	1.04 (0.99–1.08, *p* = 0.105)
Duration of hospitalization (in months; *n* = 589)	Mean (SD)	6.5 (13.1)	6.5 (14.5)	6.4 (8.8)	0.221	1.00 (0.98–1.01, *p* = 0.976)
Suicide attempt (*n* = 704)	No	493 (70.0)	367 (75.7)	126 (57.5)	**<0.001**	–
	Yes	211 (30.0)	118 (24.3)	93 (42.5)		2.30 (1.64–3.22, *p* < 0.001)
Global Assessment of Functioning (*n* = 520)	Mean (SD)	56.7 (13.4)	57.9 (13.4)	54.0 (12.9)	**<0.001**	0.98 (0.96–0.99, *p* = 0.002)
Clinical Global Impression (*n* = 516)	Mean (SD)	4.1 (1.1)	4.0 (1.1)	4.5 (0.9)	**<0.001**	1.54 (1.28–1.87, *p* < 0.001)
CASIG adaptation—satisfaction level with interpersonal relationships (*n* = 575)	Mean (SD)	5.8 (2.9)	6.4 (2.7)	4.5 (2.8)	**<0.001**	0.79 (0.73–0.84, *p* < 0.001)
CASIG adaptation—satisfaction level with family relationships (*n* = 575)	Mean (SD)	6.7 (2.6)	7.1 (2.4)	6.0 (2.7)	**<0.001**	0.85 (0.79–0.91, *p* < 0.001)
CASIG adaptation—satisfaction level with intimate relationships (*n* = 569)	Mean (SD)	3.9 (3.2)	4.4 (3.2)	3.1 (3.0)	**<0.001**	0.88 (0.83–0.93, *p* < 0.001)
CASIG adaptation—satisfaction level with vocational status (*n* = 555)	Mean (SD)	3.2 (2.9)	3.5 (3.0)	2.8 (2.7)	**0.006**	0.92 (0.86–0.98, *p* = 0.008)
CASIG adaptation—satisfaction level with education (*n* = 574)	Mean (SD)	5.2 (2.9)	5.5 (2.8)	4.6 (3.0)	**<0.001**	0.90 (0.85–0.96, *p* = 0.001)
SQoL18 total score (% satisfaction) (*n* = 655)	Mean (SD)	50.8 (17.9)	54.5 (17.3)	42.8 (16.5)	**<0.001**	0.96 (0.95–0.97, *p* < 0.001)
SERS total score (*n* = 630)	Mean (SD)	1.6 (20.7)	9.0 (18.4)	−13.7 (16.5)	**<0.001**	0.93 (0.92–0.94, *p* < 0.001)
Global Stage Of Recovery Instrument (max of stages) (*n* = 621)	5—Growth	199 (32.0)	174 (41.0)	25 (12.7)	**<0.001**	–
	1—Moratorium	123 (19.8)	51 (12.0)	72 (36.5)		9.83 (5.73–17.33, *p* < 0.001)
	2–Awareness	93 (15.0)	52 (12.3)	41 (20.8)		5.49 (3.08–9.97, *p* < 0.001)
	3—Preparation	63 (10.1)	40 (9.4)	23 (11.7)		4.00 (2.06–7.79, *p* < 0.001)
	4—Rebuilding	143 (23.0)	107 (25.2)	36 (18.3)		2.34 (1.34–4.15, *p* = 0.003)
IS Birchwood total score (*n* = 634)	Mean (SD)	8.8 (2.5)	8.4 (2.6)	9.6 (2.1)	**<0.001**	1.27 (1.17–1.38, *p* < 0.001)
Warwick-Edinburgh Mental Well-Being Scale (*z*-score; *n* = 705)	Mean (SD)	−1.2 (1.3)	−1.0 (1.3)	−1.9 (1.1)	**<0.001**	0.54 (0.47–0.62, *p* < 0.001)
Medication Adherence Rating Scale total score (*n* = 594)	Mean (SD)	6.7 (1.9)	6.9 (1.9)	6.3 (2.0)	**<0.001**	0.84 (0.77–0.92, *p* < 0.001)

Values are *n* (%) or mean (SD). Bold indicates *p* value <0.05.

In the multivariate analysis (Table 4), elevated self-stigma was associated with early stages of personal recovery (moratorium, *p* = 0.001, OR = 4.0 [1.78–8.98]; awareness, *p* = 0.011, OR = 2.87 [1.28–6.44]), history of suicide attempt (*p* = 0.001, OR = 2.27 [1.37–3.76]), insight (*p* = 0.002, OR = 1.22 [1.08–1.38]), wellbeing (WEMWBS, *p* = 0.037, OR = 0.77 [0.60–0.98]) and satisfaction with interpersonal relationships (*p* < 0.001, OR = 0.85 [0.78–0.93]).

**Table 4.** Multivariate logistic regression summary (with stepwise selection)

Predictors (*n* = 380)	Self-stigma level				
Estimate	Std. error	*p*-Value	Odds ratio	95% CI for odds ratio
(Intercept)	−2.91	0.70	**<0.001**	0.05	0.01–0.22
Suicide attempt (yes)	0.82	0.26	**0.001**	2.27	1.37–3.76
CASIG adaptation—satisfaction level in friend relationships	−0.16	0.05	**<0.001**	0.85	0.78–0.93
STORI: 1—Moratorium (ref: 5—Growth)	1.39	0.41	**0.001**	4	1.78–8.98
STORI: 2—Awareness	1.05	0.41	**0.011**	2.87	1.28–6.44
STORI: 3—Preparation	0.67	0.43	0.12	1.95	0.84–4.52
STORI: 4—Rebuilding	0.38	0.38	0.318	1.47	0.69–3.11
IS Birchwood total score	0.20	0.06	**0.002**	1.22	1.08–1.38
WEMWBS (*z*-score)	−0.26	0.12	**0.037**	0.77	0.60–0.98

Model chi-squared (8) = 104.1, *p* < 0.001. Bold values refer to statistically significant correlations.

## Discussion

To the best of our knowledge, this study is the first to assess the prevalence of self-stigma in a multicentric nonselected psychiatric rehabilitation SMI and ASD sample. As expected, the prevalence of self-stigma in France was high (31.3%). The highest proportion of individuals with elevated self-stigma was found in BPD (43.8%) and the lowest in ASD (22.2%). Self-stigma was higher in BD, MDD, and anxiety disorders compared with SZ. Female gender and older age at the time of admission predicted self-stigma in contrast with other socio-demographic variables (education level, occupational status, housing status, intimate relationships, parenting status). Illness duration, the number of psychiatric hospitalizations, a history of suicide attempt, and a greater insight into illness were significant correlates of the level of self-stigma. Other variables such as psychiatric diagnosis, age of onset, the duration of psychiatric hospitalizations and cognitive functioning did not yield significant associations. Self-stigma positively correlated with symptom severity, clinical severity, and psychiatric comorbidities. Elevated self-stigma was negatively associated with treatment adherence, psychosocial functioning, self-esteem, QoL, wellbeing, and satisfaction with different life domains and personal recovery. Self-stigma and personal recovery were strongly correlated in the multivariate model. Individuals in the early stages of personal recovery (moratorium or awareness) had a fourfold and a threefold risk, respectively, of presenting an elevated level of self-stigma compared with individuals in the growth stage. Other significant predictors of self-stigma in the multivariate model were a history of suicide attempt (twofold risk of elevated self-stigma), a higher insight into illness (risk ×1.22) and decreased wellbeing (risk ×1.3) and satisfaction with interpersonal relationships (risk ×1.18).

The prevalence of elevated self-stigma for SMI is comparable with other studies conducted in other countries across the world (weighted proportion = 31.5 of the 5,457 participants included in 27 studies [19]). Self-stigma in SZ was within the average for European countries (mean IS = 2.16 in 10 studies [19]), elevated self-stigma being less prevalent than in Brohan et al. (41.7%, mean IS = 2.40 [2]). The important country-related %variations in this study may explain these differences (from 15.2% in Sweden to 50% in Croatia [2]). Elevated self-stigma in SZ was less frequent compared with South Asia (weighted prevalence = 36.8% [19]), South-East Asia (36.6%), Africa (39.4%), North America (44.2%), and South America (38.6%) [57]. Cultural factors and country or setting-related differences might explain these variations [19]. Self-stigma in mood disorders was higher compared with other European countries (mean IS = 1.94 BD; 2.11 MDD, 21.7% with moderate-high self-stigma [3]) and Turkey (mean IS = 2.10 BD, 18.5%) [58]. Self-stigma in BD was comparable to the results of a U.S. study with nonadherent patients (mean IS = 2.22; 26% [59]). Self-stigma in anxiety disorders was higher than in the Czech Republic (mean IS = 2.24 [22]; mean IS = 1.98 [20]). There are several potential explanations of this higher prevalence of self-stigma. The subsamples of patients with MDD or anxiety disorders were small in size (*n* = 27; *n* = 19) and may not be representative. A significant proportion of patients with mood disorders included in the REHABase network had comorbid personality (14% in BD; 21% in MDD) or anxiety disorders (8% in BD, 12% in MDD [32]). In addition, the proportion of patients working in mainstream environments was very low in the REHABase network [32] compared with employment rates in BD (40–60% [60]). It can therefore be supposed that the patients with mood disorders referred to the centers for psychiatric rehabilitation had more severe psychiatric comorbidities and were more self-stigmatized than the average. The prevalence of elevated self-stigma in BPD was high, in line with other studies (mean IS = 2.45 [20]; mean IS = 2.37 [23]). Self-stigma in ASD was more frequent than in a recent German study (mean IS = 1.93, 15.4% [21]). This might be related to the levels of public stigma towards ASD in France [1]. Self-stigma should be more systematically investigated in patients with BPD or ASD [20,21].

Few socio-demographic and illness-related variables significantly predicted the level of self-stigma in our sample. The correlations with female gender and older age at the time of admission concur with certain studies [61–63] but contradict others [20,29]. According to some authors, tertiary education and employment could protect against self-stigma [2,3,61,62,64]. This was not the case in our sample, and this is consistent with other studies [59,65]. The absence of a correlation between self-stigma, housing status and intimate relationships corresponds to the findings of most studies on self-stigma [19]. Self-stigma was not associated with parenting status, in contrast to previous research [24]. Higher insight into illness significantly predicted the level of self-stigma, in accordance with several studies [11,66]. A history of suicide attempt was associated with elevated self-stigma as found in other studies [67,68]. As self-stigma moderates the relationship between insight into illness and depression, it can be supposed that recovery-oriented interventions targeting self-stigma reduction could protect against depression and suicidal ideation and should be further developed [13,14]. Illness duration and the number of psychiatric hospitalizations also predicted self-stigma. This concurs with the findings of some studies [60 but contradicts others [5,65]. Age of onset did not predict self-stigma, as in the majority of previous studies [5,59,65]. Psychiatric diagnosis did not predict the level of self-stigma, which is consistent with a large number of studies [27,28,58,64]. This contrasts with other studies, which found that self-stigma was higher in SZ [61,69], in MDD compared with BD [3], or in BPD compared with other SMI [20,22,29]. This study was the first to compare self-stigma in SMI and ASD. Elevated self-stigma was less frequent in ASD, but the mean self-stigma scores did not differ significantly from SMI. Considering the small number of articles on self-stigma in ASD, further research should investigate the relationships between public stigma, perceived stigma, experienced stigma, and self-stigma in ASD. The absence of correlation among neurocognition, social cognition, and self-stigma in our sample was unexpected, as cognitive impairments have been found to be negatively associated with self-stigma in previous studies [15–17,70]. Changes in cognition have been related to functional improvements [71]. Further research should investigate whether improvements in neurocognition and social cognition during psychiatric rehabilitation lead to a reduction in self-stigma. Elevated self-stigma was negatively associated with wellbeing and satisfaction with interpersonal relationships. This concurs with several studies, self-stigma being associated with decreased wellbeing and life satisfaction [72,73]. Self-stigma was consistently negatively associated with QoL in a large body of literature (27 studies [19]). A strong positive correlation was found between self-stigma and the early stages of personal recovery. This reflects the findings of several studies showing a negative association between self-stigma and personal recovery [10,17] (15 studies [19]). The associations between preserved insight into illness, elevated self-stigma, and the early stages of personal recovery in our sample consistently support many of the predictions of the “illness-identity model” [6].

In short, self-stigma was highly prevalent in a large nonselected sample of people with SMI and ASD enrolled in psychiatric rehabilitation. The correlations between self-stigma, insight, previous suicide attempt, wellbeing, satisfaction with interpersonal relationships, and personal recovery indicate the need to further develop recovery-oriented interventions targeting self-stigma. The effectiveness of psychiatric rehabilitation on self-stigma and the potential mediating role of changes in self-stigma on treatment outcomes should be further investigated.

### Limits

Although the REHABase network covers a large proportion of the French territory, it cannot be definitively asserted that its database constitutes a representative sample of the French population of SMI and ASD patients. The REHABase database is composed of participants enrolled in psychiatric rehabilitation and might therefore not be representative of all patients with SMI or ASD. However, some sample characteristics (including sex ratio, age at illness onset, comorbidities) suggest that the present sample is comparable to the general community-dwelling SMI population.

### Strengths

The present study has some clear strengths: a large nonselected sample of community-dwelling SMI and ASD outpatients, the use of a large bundle of standardized evaluation scales, and the inclusion of a large number of potential confounding factors in the multivariate analysis.
